# Genome Sequence of the Radiation-Resistant Bacterium Deinococcus radiophilus ATCC 27603^T^

**DOI:** 10.1128/MRA.00627-19

**Published:** 2019-07-25

**Authors:** Claudia R. Maynard, Kyle S. MacLea

**Affiliations:** a Biotechnology Program, University of New Hampshire, Manchester, New Hampshire, USA; b Biology Program, University of New Hampshire, Manchester, New Hampshire, USA; c Department of Life Sciences, University of New Hampshire, Manchester, New Hampshire, USA; Loyola University Chicago

## Abstract

The pigmented bacterium Deinococcus radiophilus, which is highly resistant to radiation exposure, was first isolated from irradiated lizardfish. We report a genome assembly of D. radiophilus UWO 1055^T^ (=ATCC 27603^T^), with a predicted genome size of 2.7 Mbp (62.66% G+C content). A number of CRISPR-associated proteins and two CRISPR arrays were identified.

## ANNOUNCEMENT

Compared to highly sequenced bacterial phyla, such as the *Proteobacteria*, to date, only about 53 genome assemblies from the phylum *Deinococcus*-*Thermus* have been made publicly available. Within the phylum, organisms of the radiation-resistant genus Deinococcus were separated from the actinobacterial genus Micrococcus (which has superficial similarities to the deinococci on the basis of pigmentation and Gram-positive staining) when the new genus was created (1). These unusual Gram-positive organisms (that also possess an outer membrane) include the most studied radiation-resistant organism, Deinococcus radiodurans (1), for which original (2) and improved (3) genome assemblies are available. Few other deinococci have available genome sequences, and there is some controversy on the relatedness of these organisms that was recognized early on by Brooks and Murray (1) and for which they suggested a possible need to split the genus at a later date. Additional genomes in this genus will help settle this and other issues.

First found in an irradiated lizardfish (the species known as Bombay duck or bummalo [Harpadon nehereus]), *Deinococcus radiophilus* is an obligate aerobic deinococcus, stains Gram positive, and is pigmented and non-spore forming. *D. radiophilus* (4–7) differs from D. radiodurans and other members of the genus by a lack of nitrate reduction and no production of acid from glucose in standard media (1). *D. radiophilus* is also of note because of its remarkable DNA repair abilities and its production of useful restriction endonucleases (8, 9). There is also some interest in the use of deinococci, including *D. radiophilus,* in the bioremediation of accidents involving radioactive materials and in novel biotechnology applications (10, 11).

Freeze-dried *D. radiophilus* ATCC 27603^T^ was rehydrated and streaked for single colonies on Trypticase soy agar at 30°C for 48 h at 1 atm. A Trypticase soy broth culture was grown from a single colony and used to isolate genomic DNA (gDNA) by means of the QIAamp DNA minikit (Qiagen, Valencia, CA, USA). The Kapa HyperPlus kit (KR1145, v.3.16; Wilmington, MA, USA) was used to fragment gDNA and tag it with sequence adapters for sequencing on an Illumina HiSeq 2500 instrument (Hubbard Center for Genome Studies, Durham, NH, USA). A total of 4,543,128 raw 250-bp reads were trimmed using Trimmomatic (settings, paired-end mode with a window size of 4, quality requirement of 15, and minimum read length of 36) before being assembled with the default parameters for bacterial assembly using SPAdes v.3.11.1 (12, 13). Once small contigs (<500 bp) and contaminants (flagged by the NCBI using the PGAP, described below) were removed, QUAST (14) identified 100 contigs, with the largest one being 325,917 bp, with an *N*_50_ value of 77,319 bp and an estimated genome coverage of 754×. Annotation was conducted using the NCBI Prokaryotic Genome Annotation Pipeline (PGAP) (15). PGAP revealed a total sequence length of 2,753,082 bp, a total of 2,789 genes, 2,731 protein-coding sequences, 78 pseudogenes, 50 tRNAs, 5 rRNAs, 3 noncoding RNAs (ncRNAs), 2 CRISPR arrays, and a G+C content of 62.66%, in line with the published value (determined by the melting temperature [*T_m_*]) of 62% (1).

### Data availability.

The Deinococcus radiophilus ATCC 27603^T^ whole-genome shotgun sequence (WGS) project has been deposited at DDBJ/ENA/GenBank under the accession number RXPE00000000. The version described in this paper is version RXPE01000000. The raw Illumina data from BioProject number PRJNA509618 were submitted to the NCBI Sequence Read Archive (SRA) under experiment accession number SRX5889021.
